# Elevated cytokine levels in tears and saliva of patients with primary Sjögren’s syndrome correlate with clinical ocular and oral manifestations

**DOI:** 10.1038/s41598-019-43714-5

**Published:** 2019-05-13

**Authors:** Xiangjun Chen, Lara A. Aqrawi, Tor Paaske Utheim, Behzod Tashbayev, Øygunn Aass Utheim, Sjur Reppe, Lene Hystad Hove, Bente Brokstad Herlofson, Preet Bano Singh, Øyvind Palm, Hilde Kanli Galtung, Janicke Cecilie Liaaen Jensen

**Affiliations:** 1 Department of Oral Surgery and Oral Medicine, Faculty of Dentistry, University of Oslo, Oslo, Norway; 2 The Norwegian Dry Eye Clinic, Oslo, Norway; 3 Department of Oral Biology, Faculty of Dentistry, University of Oslo, Oslo, Norway; 4 Department of Medical Biochemistry, Oslo University Hospital, Oslo, Norway; 5 Department of Cariology and Gerodontology, Faculty of Dentistry, University of Oslo, Oslo, Norway; 6 Department of Rheumatology, Oslo University Hospital, Oslo, Norway

**Keywords:** Diagnostic markers, Autoimmune diseases

## Abstract

Investigating cytokines in tear fluid and saliva may offer valuable information for understanding the pathogenesis of primary Sjögren’s syndrome (pSS). Cytokine profiles in both tear fluid and saliva of pSS patients, non-Sjögren’s syndrome (non-SS) subjects with sicca symptoms, and healthy controls without sicca complaints were analysed. Furthermore, relationships associating the severity of clinical ocular and oral manifestations with the upregulated cytokines were assessed. In tear fluid, pSS patients showed elevated levels of IL-1ra, IL-2, IL-4, IL-8, IL-12p70, IL-17A, IFN-γ, IP-10, MIP-1b, and Rantes compared to non-SS subjects and healthy controls. The increased cytokine levels (except IP-10) correlated significantly with reduced tear production, less stable tear film, and greater ocular surface damage. In saliva, pSS patients had a higher IP-10 level, which correlated with higher candida score; and an elevated MIP-1a level, which correlated significantly with lower unstimulated and stimulated whole saliva secretion rates. The upregulated cytokines identified in tear fluid and saliva of pSS patients show a clear interplay between innate and adaptive immune responses that may contribute to disease pathogenesis. The increase of IP-10 and MIP in both tears and saliva further emphasises the essential role of macrophages and innate immunity in pSS.

## Introduction

Sjögren’s syndrome (SS) is a systemic rheumatic and autoimmune disorder characterised by lymphocytic infiltration of the exocrine glands in multiple sites, particularly the salivary and lacrimal glands^1^. Clinically, SS may affect multiple organ systems including the exocrine glands in the skin, respiratory, urogenital, and gastrointestinal tracts, as well as having extra-glandular involvement^2^. It is considered primary (pSS) when it arises alone, and secondary (sSS) when occurring in association with other underlying autoimmune diseases, such as rheumatoid arthritis and systemic lupus erythematosus^3^. The involvement of lacrimal and salivary glands may lead to qualitatively altered and diminished lacrimal and salivary secretion, eventually resulting in the common symptoms of dry eye (xerophthalmia) and dry mouth (xerostomia)^1^. Studies have shown that these sicca symptoms are also associated with fatigue, depression, and quality of life impairments in patients with SS^4,5^.

Despite decades of research, the pathogenic mechanisms associated with dry eyes and dry mouth in pSS remain incompletely understood. Moreover, clinical tools for measuring ocular and oral signs are often susceptible to subjective interpretation and dependent on the investigator’s clinical experience. Meanwhile, protein analysis has the advantage of being less prone to subjective bias^6^. The characteristic histological feature of pSS is infiltration of mononuclear cells into exocrine glands. These include CD4+ T cells, B cell subsets, dendritic cells and macrophages, which contribute to the dysfunction and eventually destruction of the exocrine glands by initiating an inflammatory response^7–10^. Inflammatory cytokine levels are hence expected to be elevated in fluid from affected glands, such as salivary and tear fluid. Therefore, tear fluid and saliva may represent a vital experimental source containing valuable biomarkers for diagnostic and therapeutic purposes in pSS^11,12^. Advances in the technology of multiplex bead arrays have allowed this technique to be used in detecting proteins of low abundance in small sample volumes. Indeed, the multiplex immunoassays have been employed to investigate cytokine levels in saliva^13,14^ and tear fluid^15,16^ of patients with SS, and demonstrated consistency with ELISA assay findings^17^.

Several multiplex bead-based immunoassay studies have previously identified a variety of elevated proinflammatory cytokines in tear- and salivary fluid of patients with pSS compared to healthy controls^13–15,17^. Such cytokines include IL-4, IL-6, IL-8, IL-10, IL-12p70, IL-17, TNF-a in tear fluid^15,17^, and MIP-1a (CCL3), MIP-1b (CCL4), IL-8 (CXCL8), IFN-r, TNF-a, IL-1, IL-4, IL-6, IL-10, IL-12p40, and IL-17 in saliva^13,14^. Additionally, the increase in cytokine levels has also been shown to directly correlate with clinical dry eye and dry mouth manifestations in these subjects^13–15,18^. Moreover, previous studies have investigated cytokine levels of non-SS sicca controls^13,14^. These individuals suffer from dry eye and dry mouth symptoms, but do not satisfy the diagnostic criteria for SS. Nonetheless, an increase in proinflammatory cytokines, for instance, IL-2, IL-4, IL-6, IL-8, IL-17, and TNF-a in tear fluid^15,17,18^, and salivary MIP-1a (CCL3), IL-8 (CXCL8), IP-10 (CXCL10), and TNF-a^13,14^ in pSS patients, compared to that of non-SS subjects, was also observed in these instances.

In spite of the large repertoire of studies to date exploring cytokine expression in patients with pSS, none of them simultaneously explored and compared cytokine levels in both tear- and salivary fluid from the same individuals. Hence, in the current study we analysed cytokine profiles in both tear fluid and saliva in the same cohort of pSS patients, and compared with both age- and gender-matched healthy controls and non-SS sicca subjects, in the search of tear and saliva pSS specific markers. We further investigated whether the array of upregulated cytokines in tear fluid and saliva correlated with the severity of clinical ocular and oral symptoms and manifestations.

## Methods

### Study participants

In the present study, 29 female pSS patients were recruited at the Department of Rheumatology, Oslo University Hospital, Norway. The pSS patients fulfilled the classification criteria of pSS according to the American-European Consensus Group (AECG)^1^. In order to obtain a homogenous patient group, all patients were required to have Sjögren’s syndrome-related anti-SSA autoantibodies in serum. In addition, 20 age- and gender- matched non-SS sicca control subjects, and 17 healthy control subjects were recruited at the Faculty of Dentistry, University of Oslo, Norway. The patients in the non-SS group all had dry eye and dry mouth symptoms and were previously referred to the Institute of Clinical Odontology, Faculty of Dentistry, University of Oslo for minor salivary gland biopsy (JLJ). However, they did not fulfil the classification criteria for pSS as they were anti-SSA/SSB negative and had demonstrated a focus score <1 in their salivary gland biopsy. The demographic data obtained through clinical examination and from patients’ charts at the Department of Rheumatology, Oslo University Hospital for the pSS and non-SS patients included in the current study are presented in Table 1. Some of the pSS patients and non-SS subjects used artificial tears for dry eyes, but none of the participants used topical anti-inflammatory drugs such as steroids or cyclosporine. The inclusion criteria for the healthy controls included no complaints of dryness in the mouth or eyes, dry eye severity level (DESL) ≤ 1 or unstimulated whole saliva (UWS) flow rate of >0.1 ml/min, absence of systemic disorders with oral or ocular involvement, and no history of surgical procedures that might affect secretion from the lacrimal and salivary glands. The mean age was 56.8 ± 13.0 years (mean ± SD), 51.7 ± 10.6 years, and 45.4 ± 10.9 years in the pSS, non-SS sicca control, and healthy control groups, respectively.

**Table 1:** Clinical characteristics of pSS and non-SS patients included in the study.

Study ID	Age (years)	Anti-SSA*	Anti-SSB*	Focus score**	Schirmer’s test***	Saliva secretion****	Dry Eyes	Dry Mouth
pSS1	39	+	+	NA	+	+	+	+
pSS2	54	+	−	NA	+	+	+	+
pSS3	36	+	−	NA	+	+	+	+
pSS4	53	+	−	NA	+	+	+	+
pSS5	72	+	−	NA	+	−	+	+
pSS6	68	+	+	NA	+	+	+	+
pSS7	48	+	+	NA	+	+	+	+
pSS8	48	+	+	NA	+	+	+	+
pSS9	57	+	+	NA	+	+	+	+
pSS10	35	+	+	NA	+	+	+	+
pSS11	71	+	−	NA	+	+	+	+
pSS12	48	+	−	NA	+	+	+	+
pSS13	47	+	+	NA	+	+	+	+
pSS14	64	+	+	NA	+	+	+	+
pSS15	64	+	+	6	+	+	+	+
pSS16	59	+	+	NA	+	+	+	+
pSS17	56	+	+	8	+	+	+	+
pSS18	57	+	−	0	+	+	+	−
pSS19	75	+	−	0	+	−	+	NA
pSS20	75	+	−	0	+	+	+	+
pSS21	64	+	+	3	+	+	−	−
pSS22	73	+	−	0	+	+	+	+
pSS23	34	+	−	0	+	+	+	−
pSS24	65	+	−	0	+	+	+	+
pSS25	56	+	−	1	+	+	+	+
pSS26	49	+	+	NA	+	−	+	+
pSS27	68	+	+	NA	−	+	+	+
pSS28	36	+	+	3	+	+	−	−
pSS29	75	+	+	NA	+	−	+	+
nonSS1	42	−	−	0	+	+	+	+
nonSS2	34	−	−	0	−	+	+	+
nonSS3	56	−	−	0	+	+	+	+
nonSS4	53	−	−	<1	+	+	+	+
nonSS5	45	−	−	<1	−	−	+	+
nonSS6	46	−	−	0	−	+	+	+
nonSS7	65	−	−	<1	+	+	+	+
nonSS8	42	−	−	0	+	+	+	+
nonSS9	52	−	−	0	+	+	+	+
nonSS10	56	−	−	<1	+	+	+	+
nonSS11	58	−	−	0	+	−	+	+
nonSS12	68	−	−	0	+	+	+	+
nonSS13	76	−	−	<1	+	+	+	+
nonSS14	38	−	−	0	+	+	+	+
nonSS15	51	−	−	0	+	+	+	+
nonSS16	60	−	−	<1	+	+	+	+
nonSS17	48	−	−	0	+	−	+	+
nonSS18	44	−	−	<1	−	−	+	+
nonSS19	43	−	−	0	+	−	+	+
nonSS20	57	−	+	0	+	−	+	+

NA = data not available.

*Autoantibody production was assessed by ELISA.

**Values are the number of focal infiltrates/4 mm^2^ tissue area containing >50 mononuclear cells.

***Values are in mm/5 minutes; normal flow >5 mm/5 min. “+” indicates dryness and tear secretion ≤5 mm/5 minutes.

****Values are in ml/15 minutes; normal flow >1.5 ml/15 minutes. “+” indicates dryness and unstimulated whole saliva secretion ≤1.5 ml/15 minutes.

The study protocol was approved by the Regional Medical Ethical Committee of South-East Norway (REK 2015/363). Moreover, the study was performed in compliance with the tenets of the Declaration of Helsinki. The study subjects were referred to the Dry Mouth Clinic, Faculty of Dentistry, University of Oslo, and the Norwegian Dry Eye Clinic in Oslo for thorough examination and sample collection, as described below. The study protocol was explained to the participants, and written informed consent was obtained from all subjects prior to clinical testing.

### Dry eye and dry mouth clinical evaluation, sample collection, and storage

The clinical evaluation of dry eyes and dry mouth was performed as previously described^19,20^. Briefly, participants were asked to avoid using any eye drops at least two hours before their visit to the Norwegian Dry Eye Clinic, and not to have anything in the mouth for at least one hour before their visit to the Dry Mouth Clinic. At the Norwegian Dry Eye Clinic, the severity of dry eye symptoms was evaluated using the Ocular Surface Disease Index (OSDI) questionnaire, followed by: (1) tear film osmolarity measurement using TearLab Osmolarity System (TearLab Corp, San Diego, CA, USA); (2) tear quality evaluation using tear film break-up time (TBUT) after instillation of 5 µl 2% fluorescein sodium; (3) grading of corneal and bulbar conjunctival staining with fluorescein recorded according to the Oxford scoring scheme [range of ocular surface staining (OSS): 0–15; range of corneal staining (CS): 0–5]^21^; (4) measurement of tear production using Schirmer’s test without topical anesthesia; (5) assessment of the meibomian gland (MG) functionality; and (6) meibography images obtained using the non-contact infrared camera system Oculus Keratograph 5 (Oculus, Wezlar, Germany) after eyelids were everted. Assessment of the MG functionality was conducted by application of light pressure using cotton tips on the central five MGs of the lower eyelid. Expression was recorded as the number of the MGs with meibum secretion under pressure. The quality of the meibum that was secreted from each gland was graded as following: 0, clear; 1, cloudy; 2, cloudy with particles; and 3, toothpaste like. The average meibum quality value per expressible meibomian gland per eyelid was recorded. Based on meibography, meibomian gland dropout scores (MGDS) in both upper lid (MGDS_UL) and lower lid (MGDS_LL) were calculated as the percentage area of MG loss in relation to the total visible tarsal area: a score of 1 represented 0–25% area of MG loss; a score of 2: 26–50%; a score of 3: 51–75%; and a score of 4: > 75%. Based on the above mentioned tests, dry eye severity level (DESL) ranging from 0–4 was determined according to the standard DESL scheme^22^.

Tear fluid was collected with the Schirmer’s tear test strip (HAAG-STREIT, Essex, UK) during the Schirmer’s test. Each Schirmer strip was transferred to an eppendorf tube containing 500 µl of 0.1 µm filtered phosphate-buffered saline (PBS) pH 7.4 (Gibco, ThermoFisher Scientific, Oslo, Norway), and then stored at −80 °C until cytokine analysis. In the current study, data from the right eye was used for analysis.

At the Dry Mouth Clinic, the Summated Xerostomia Inventory – Dutch Version (SXI-D)^23^ questionnaire with a score ranging from 5 to 15 was used to determine the severity of subjective feelings of xerostomia, where maximum score indicates that the participant is experiencing the most severe problems related to dry mouth. The Clinical Oral Dryness Score index (CODS), determined from 10 different features of oral dryness, was used to acquire an objective clinical score for oral dryness, ranging from 0 to 10^24^, with higher scores indicating greater severity of mouth dryness. The presence of oral candida was tested by rubbing a sterile cotton swab over the left cheek and the anterior part of the tongue. Samples were inoculated and incubated, and growth was then semi-quantitatively scored from 0 to 3: 0 = no growth, 1 = minimal growth, 2 = moderate growth, and 3 = severe growth. Unstimulated-(UWS) and chewing-stimulated whole saliva (SWS) were collected to determine saliva secretion rates. Due to the limited amount of UWS pSS patients could produce, chewing-stimulated whole saliva sample were used for the cytokine analyses. This was aliquoted and stored at −80 °C until further analysis.

### Analyses of the saliva and tear fluid

Cytokine concentration in the tear fluid and saliva was measured using immunoassay technology (Bio-Plex XMap; Bio-Rad Laboratories, Inc., Hercules, CA, USA) with the commercial instrument Luminex IS 100 (Luminex Corp., Austin, TX, USA) powered by the Bio-Plex Software version 6.0.1 (Bio-Rad Laboratories, Inc., Hercules, CA, USA). Nineteen pSS patients, 13 healthy controls, and 16 non-SS subjects had multiplex assay analyses performed on both tear fluid and saliva, whereas the rest had multiplex assay analyses either on tear fluid or saliva. Prior to analysis, eppendorf tubes with Schirmer strips stored in PBS were thawed on ice, vortexed, and 105 µl of the samples were transferred into fresh tubes. In parallel, saliva samples were centrifuged at 8000 g for 5 min at 4 °C, and 105 µl of the supernatant were also transferred into fresh tubes.

The multiplex assay analysis was performed according to a previously published protocol^25^. In brief, eppendorf tubes with tear and saliva samples were centrifuged (10 000 g for 10 min at 4 °C), one part of the supernatant was used for protein measurement, while an equal volume of PBS/1% bovine serum albumin (BSA) was added to the remaining sample. The broad screening kit used for the analysis (Bio-Plex Pro Human Cytokine 27-plex Assay, Cat. No. M50-0KCAF0Y, Bio-Rad Laboratories, Inc.) contained 27 different cytokines. These included: interleukin 1b (IL-1b), IL-2, IL-4, IL-5, IL-6, IL-7, IL-8, IL-9, IL-10, IL-12p70, IL-13, IL-15, IL-17A, IL-1 receptor antagonist (IL-1ra), eotaxin, basic fibroblast growth factor (bFGF/FGF2), interferon gamma (IFN-γ), interferon gamma-induced protein 10 (IP-10), monocyte chemoattractant protein 1 (MCP-1), granulocyte colony-stimulating factor (G-CSF), granulocyte macrophage colony-stimulating factor (GM-CSF), macrophage inflammatory protein 1α (MIP-1α/CCL3), MIP-1b, platelet-derived growth factor bb (PDGF-BB), regulated-on-activation normal T cell expressed and secreted (Rantes), tumor necrosis factor alpha (TNF-α), and vascular endothelial growth factor (VEGF). Twenty-five μL of the resulting PBS/0.5% BSA suspension was added directly to the plate for analysis.

All values obtained from the assay were in an acceptable range according to recommendations from the manufacturer (intra-percent coefficient of variation <11 and inter-percent coefficient of variation >21). Total protein concentrations in the Schirmer strip suspensions and saliva were estimated using the Pierce BCA Protein Assay Kit (Thermo Scientific, Rockford, IL, USA) and were expressed as ng/µl. The levels of cytokines were adjusted with total protein concentration and expressed as (pg of cytokine)/(mg of total protein). Moreover, in the healthy control group, six subjects had DESL  0, whereas nine subjects had DESL 1. The average protein adjusted cytokine level in the eyes with DESL 0 was used as baseline to calculate relative cytokine levels in tears. Similarly, the average protein-adjusted cytokine level in saliva in the healthy control group was used as baseline to calculate relative cytokine levels in saliva.

### Statistical analyses

Statistical analyses were performed using SPSS software version 25.0 (IBM Corporation, Armonk, NY, US.). Reported results were presented as means ± standard deviation for clinical tests, and means ± standard error for cytokine levels. The Shapiro-Wilk test was used to test the normality of the variables. Furthermore, the Kruskal-Wallis U test was used in the intergroup comparison, while the Mann-Whitney U test was applied to determine whether there was any statistical significance between two groups. Association of cytokine levels against numerical clinical parameters was performed using Spearman rank correlation. In all analyses, a *p*-value of <0.05 was considered significant.

## Results

### Clinical ocular and oral examinations demonstrate increased dry eye and dry mouth manifestations in pSS patients

**Figure 1:**
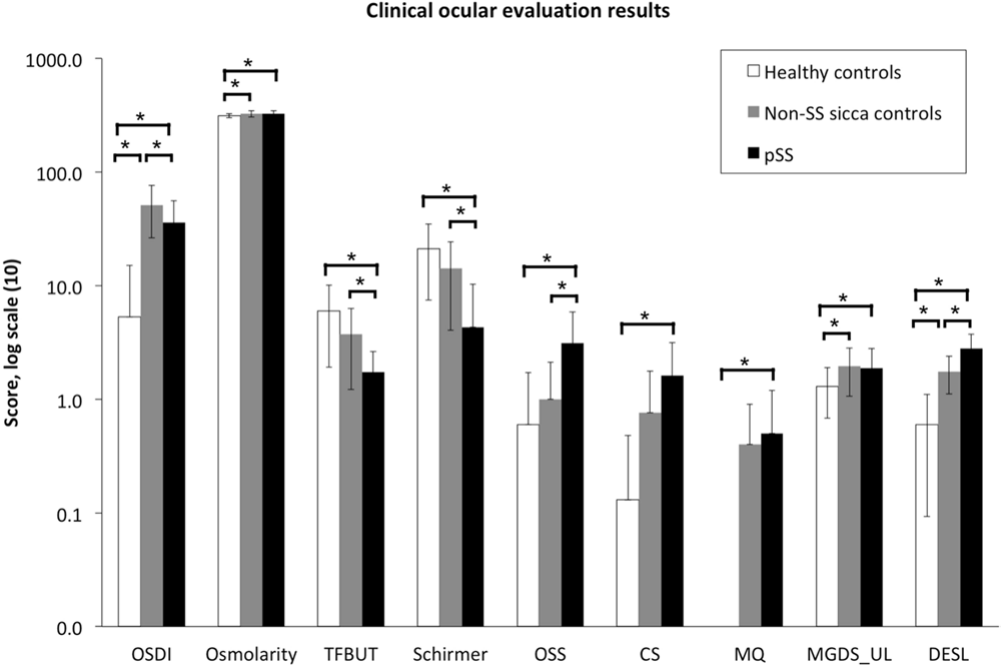
Ocular examination of the healthy control, non-SS sicca control, and pSS groups. OSDI: Ocular Surface Disease Index questionnaire score; TFBUT: tear film breakup time; OSS: ocular surface staining; CS: corneal staining; MQ: meibum quality; MGDS: meibomian gland dropout score; UL: upper lid; DESL: dry eye severity level. Intergroup difference is significant at *p < 0.05. Results are shown in the log scale, and the exact values of variables are given in the following order: healthy controls, non-SS sicca controls, and pSS. OSDI (5.3; 51.4; 36.2), Osmolarity (313.6; 327.1; 327.8), TFBUT (6.0; 3.8; 1.7), Schirmer’s test (21.2; 14.2; 4.3), OSS (0.6; 1.0; 3.1), CS (0.1; 0.8; 1.6), MQ (0.0; 0.4; 0.5), MGDS_UL (1.3; 2.0; 1.9), DESL (0.6; 1.8; 2.8).

When conducting the dry eye evaluation, the non-SS sicca controls had the most severe subjective dry eye symptoms, as shown by OSDI score. This was followed by pSS patients and healthy controls, in descending order. Tear osmolarity levels were higher in the pSS and non-SS sicca groups compared to the healthy controls. In comparison with both non-SS subjects and healthy controls, the pSS patient group had less stable tear film, demonstrated by shorter TFBUT, lower tear production levels measured with Schirmer’s test, and more severe ocular surface damage, manifested as higher ocular surface staining score. Also, pSS patients had a more severe corneal staining (1.6 ± 1.5 vs. 0.1 ± 0.4, *p* = 0.003), and a compromised meibum quality (0.5 ± 0.7 vs. 0.0 ± 0.0, *p* = 0.041) compared to healthy controls. Moreover, pSS patients and non-SS subjects had higher MGDS than healthy controls in the upper lid. No statistically significant inter-group difference was found in meibum expressibility and MGDS in the lower eyelid. Overall, the DESL was highest in the pSS patients, followed by the non-SS individuals, and finally the healthy controls (scores: 2.8 ± 0.9, 1.8 ± 0.6, and 0.6 ± 0.5, respectively) **(**Fig. 1**)**.

**Figure 2:**
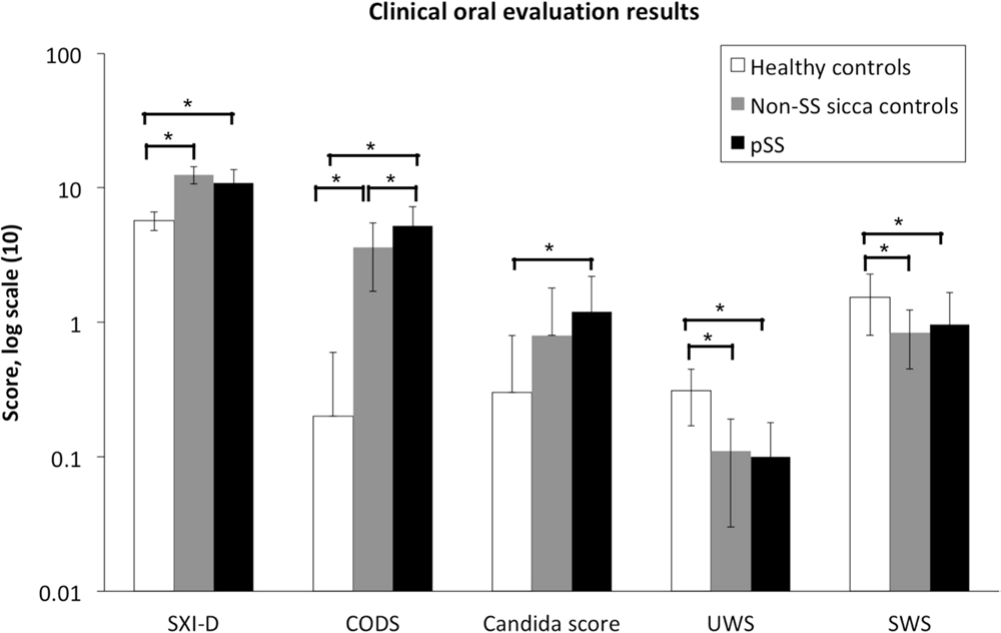
Clinical oral evaluation for the healthy control, non-SS sicca control, and pSS groups. SXI-D: Summated Xerostomia Inventory – Dutch version questionnaire score; CODS: Clinical Oral Dryness Score; UWS: unstimulated whole saliva secretion rate (ml/min); SWS: stimulated whole saliva secretion rate (ml/min). Intergroup difference is significant at *p < 0.05. Results are shown in the log scale, where exact values of variables are given in the following order: healthy controls, non-SS sicca controls, and pSS. SXI-D (5.7; 12.5; 10.9), CODS (0.2; 3.6; 5.2), Candida score (0.3; 0.8; 1.2), UWS (0.31; 0.11; 0.10), SWS (1.54; 0.84; 0.96).

Moreover, the present study demonstrated more pronounced subjective oral complaints in the pSS group and non-SS group compared to the healthy controls, presented as higher SXI-D questionnaire scores. Additionally, a significantly higher mean oral dryness score was shown in the pSS patients compared to the non-SS subjects and the healthy controls, according to the CODS index^24^. Compared to healthy controls, the pSS and non-SS groups had lower UWS and SWS. Candida counts were higher among the pSS patients compared to the healthy control group (scores: 1.2 ± 1.0 vs. 0.3 ± 0.5; *p* = 0.009). However, there were no significant differences in SXI-D scores, candida scores, UWS, or SWS between pSS and non-SS sicca control groups (*p* = 0.114, 0.246, 0.609, and 0.872, respectively) (Fig. 2).

### Elevated levels of pro-inflammatory cytokines identified in both tear fluid and saliva of pSS patients

**Figure 3:**
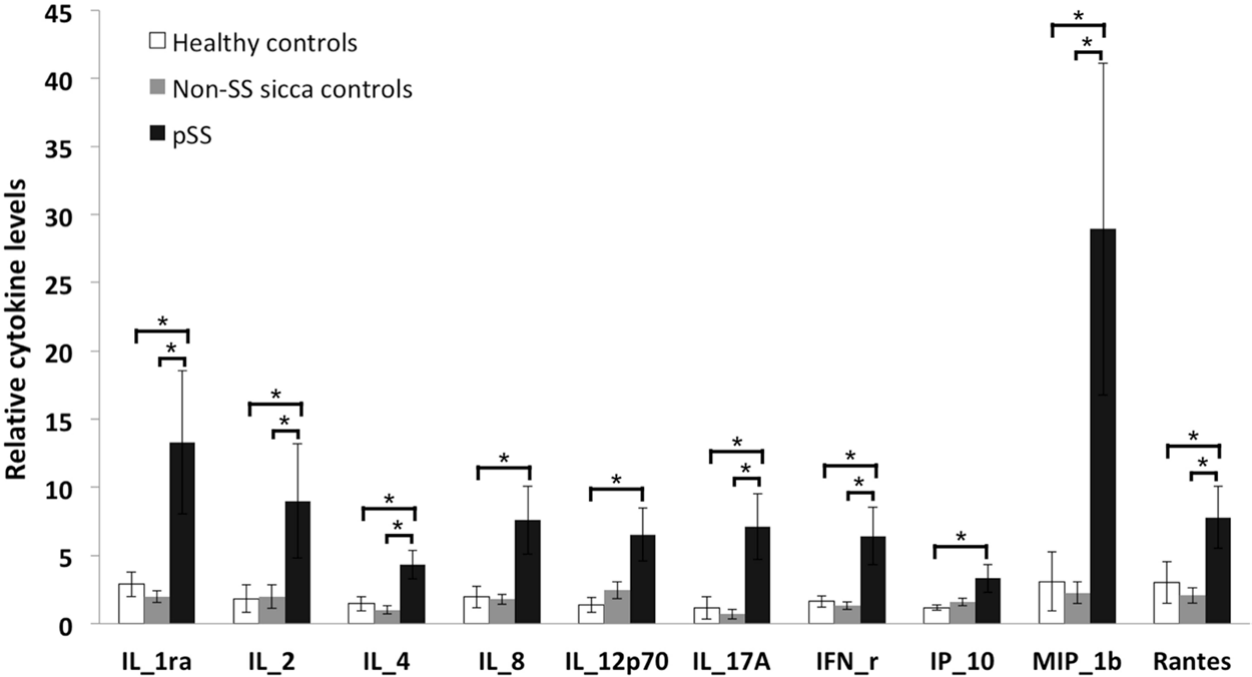
Comparison of relative cytokine levels in tear fluid. Intergroup difference is considered significant at *p < 0.05.

In tear fluid, pSS patients had higher cytokine levels than both non-SS subjects and healthy controls regarding IL-1ra, IL-2, IL-4, IL-17A, IFN-γ, MIP-1b, and Rantes. Additionally, IL-8, IL-12p70, and IP-10 levels were also higher in pSS patients compared to healthy controls. However, no significant differences were found between pSS patients and non-SS sicca controls concerning these cytokines (Fig. 3). The remaining analysed cytokines in tear fluid did not show statistically significant differences between pSS patients and healthy controls (Fig. S1).

**Figure 4:**
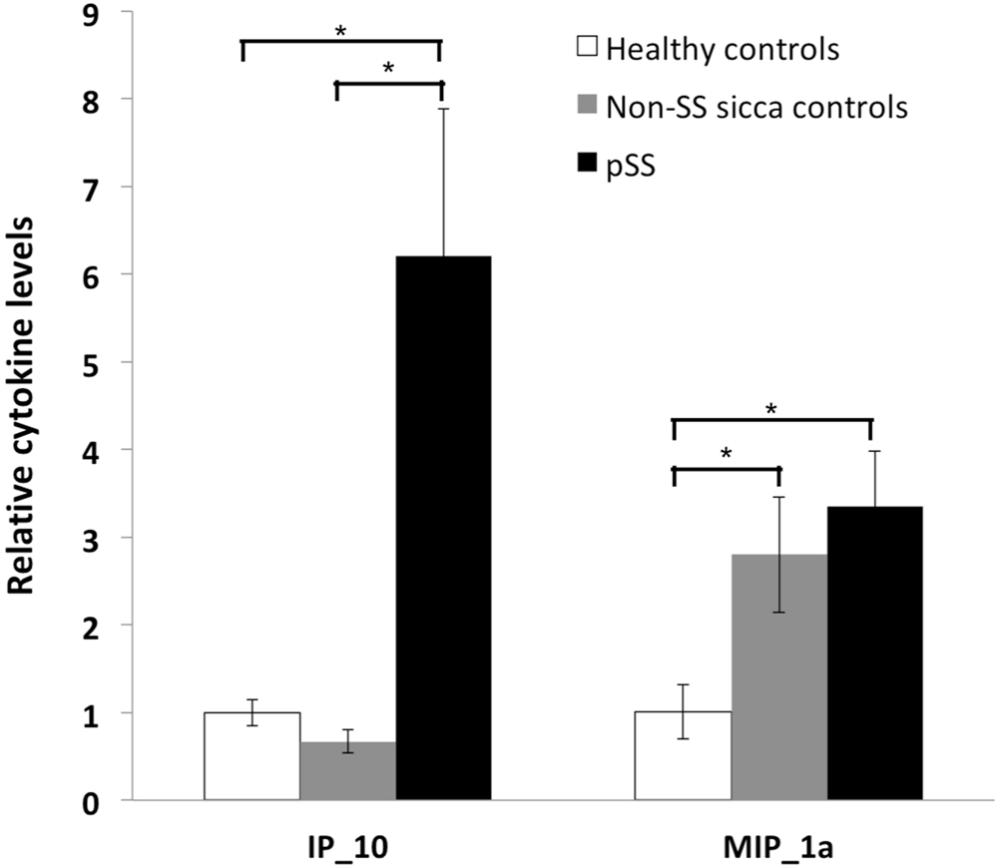
Comparison of relative cytokine levels in saliva. Intergroup difference is considered significant at *p < 0.05.

In saliva, pSS patients had a higher IP-10 level than non-SS individuals and healthy controls. Both pSS patients and non-SS subjects had higher MIP-1α levels than healthy controls (Fig. 4). The remaining analysed cytokines in SWS did not show statistically significant differences between pSS patients and healthy controls (Fig. S2).

### Distinct correlation observed between elevated pro-inflammatory cytokine levels and clinical ocular and oral parameters

Significantly elevated cytokines in tear fluid (IL-1ra, IL-2, IL-4, IL-17A, IFN-γ, MIP-1b, Rantes, IL-8, IL-12p70, and IP-10) and saliva (IP-10 and MIP-1a) in pSS patients, when compared to healthy controls, were included in the correlation analyses. The level of MIP-1b in tear fluid and saliva correlated positively with each other (r = 0.408, *p* = 0.003), the rest did not show significant correlation between tear fluid and saliva. We further calculated the correlations of these cytokine levels with the above-mentioned clinical oral and ocular findings (Table 2, only data with statistical significance are listed), respectively.

**Table 2:** Correlations identified between levels of cytokines in tear fluid and clinical ocular parameters.

Clinical Parameters		IL_1ra	IL_2	IL_4	IL_8	IL_12p70	IL_17A	IFN_y	MIP_1b	Rantes
Age	*r*				0.261*					
Osmolarity	*r*	0.389*	0.265*	0.274*	0.454**			0.354*	0.421*	0.406*
TFBUT	*r*	−0.388*		−0.349*	−0.340*	−0.280*		−0.362*	−0.315*	−0.405*
Schirmer I	*r*	−0.629**	−0.463**	−0.579**	−0.494**	−0.459**	−0.462**	−0.629**	−0.626**	−0.727**
OSS	*r*	0.443**	0.359*	0.380*	0.440**	0.333*	0.315*	0.445**	0.560**	0.533**
CS	*r*	0.313*		0.310*	0.481**	0.264*		0.360*	0.538**	0.522**
DESL	*r*	0.513**	0.392*	0.490**	0.474**	0.414*	0.380*	0.546**	0.550**	0.637**

TFBUT: tear film breakup time; OSS: ocular surface staining; CS: corneal staining; DESL: dry eye severity level. Correlation coefficient r was tested with Spearman correlation test, statistical significant is set at *p < 0.05 level and **p < 0.001.

Increased dry eye severity level and ocular surface staining correlated with increased cytokine levels, except for IP-10. Considering the relationship between tear production and cytokine levels, negative correlations were found between the Schirmer’s test results and cytokine levels for nine cytokines included in the analysis (IL-1ra, IL-2, IL-4, IL-8, IL-12p70, IL-17A, IFN-γ, MIP-1b, and Rantes) (Table 2).

Negative correlations were found between TFBUT and cytokine levels of IL-1ra, IL-4, IL-8, IL-12p70, IFN-γ, MIP-1b, and Rantes. However, no significant correlation was detected between tear cytokine levels and meibomian gland functionality evaluated by meibum expressibility and quality, or meibomian gland morphological changes observed with meibomian gland drop out scores.

In view of the relationships between salivary cytokine levels of IP-10 and MIP-1a and dry mouth evaluation results, a higher candida score correlated with an elevated IP-10 level (r = 0.295, *p* = 0.032). Meanwhile, a higher score of SXI-D correlated with raised MIP-1a (r = 0.282, *p* = 0.041), whereas UWS and SWS correlated negatively with MIP-1a level (r = −0.276, *p* = 0.046; and r = −0.282, *p* = 0.040, respectively).

## Discussion

Identifying protein biomarkers in tear fluid and saliva is desirable when studying local and systemic diseases, as sampling of tear fluid and saliva is usually manageable, inexpensive, and non-invasive. Hence, the discovery of saliva- and tear- based molecular and immunological biomarkers in SS will enable their application in disease diagnostics, as well as aid in establishing targeted therapy^11^. Accordingly, the present study showed that pSS patients had a different inflammatory cytokine profile in both tear fluid and saliva, when compared to that of healthy individuals and non-SS sicca subjects. Among these, levels of IP-10 and MIP were elevated in both tear fluid and saliva, indicating that these proinflammatory proteins may affect both organs. Correlation analyses further demonstrated associations between the levels of upregulated cytokines and clinical dry eye and dry mouth testing parameters. Such parameters included tear film osmolarity, TFBUT, Schirmer’s test, OSS, CS, DESL, oral candida score, UWS, and SWS, reflecting the clinical relevance of these cytokines in pSS.

Dry eye disease is a multifactorial disease of the ocular surface, characterised by the loss of homeostasis in the tear film, i.e., the protective layer surrounding the eye. It is also associated with ocular surface inflammation and damage^26^. Previous studies have shown that SS patients often suffer from dry eye disease, where large numbers of activated CD4+ T helper cells were found in the conjunctival tissue of the eye, indicating T-cell homing that further facilitates immune activation in the conjunctiva^27,28^. Furthermore, the secretion of proinflammatory cytokines by these activated CD4+ T helper cells in the conjunctival tissue of the eye may in turn lead to ocular surface damage through Th1- and Th2- mediated immune reactions. Indeed, in the current study, our results revealed elevated levels of Th1 type cytokines, namely IL-2, IFN-γ, IL-12, and IP10, and Th2 type cytokine IL-4, in the tear fluid of pSS patients, when compared to both non-SS sicca subjects and healthy individuals. Moreover, IL-2 and IFN-γ are known to be secreted predominantly by Th1 cells. Further, IFN-γ may also be secreted by natural killer cells, monocytes, and macrophages. This increased production of IFN-γ has previously been associated with SS^29^, where IFN-γ may also interact with epithelial tissue and propagate inflammation^30^. Similarly, in the conjunctiva, IFN-γ expression has been shown to be significantly higher in the dry eyes of SS patients, which correlated with goblet cell loss and reduced mucin production^31^. It could also play a vital role in corneal epithelial apoptosis and tissue destruction via activation of the extrinsic apoptotic pathway^32^. Meanwhile, IL-12 is mainly produced by macrophages and dendritic cells, and has been recognized to play an important role in regulating IFN-γ production and promoting the development of Th1 responses^33^. In consistence with a study by Zhao and associates^17^, our results showed increased tear IL-12p70 in SS patients compared to non-SS subjects and healthy controls. However, IL-4, also upregulated in this present study, is primarily involved in Th2-mediated immune responses and the activation of B cells^34,35^. Together, these cytokines play a critical role in both innate and adaptive immunity, and may in turn influence disease progression in these patients.

Interestingly, when compared to both non-SS subjects and healthy controls, we found significant elevation in tear MIP-1b, Rantes, IP-10, and IL-8 in pSS patients. These cytokines can attract Th1 cells, natural killer cells, macrophages, and dendritic cells that express specific receptors, such as CCR5 and CXCR3, to the corneal and conjunctival tissues. Previous studies have demonstrated that the levels of MIP-1a, MIP-1b, Rantes, and IP-10 in tear fluid increased significantly in dry eye patients, especially in those with SS, compared with healthy controls^17,36–38^. These studies also showed upregulation in the expression of MIP-1a, MIP-1b, Rantes, and their receptor CCR5, as well as the expression of IP-10 and its receptor CXCR3 in the conjunctiva. Furthermore, IL-8 has a potent chemotactic effect to T cells, neutrophils, and eosinophils^39,40^. This massive infiltration and activation of T lymphocytes may thus lead to the damage of the lacrimal gland and ocular surface tissues through cytotoxicity and apoptosis. Our finding of increased tear IL-8 in pSS patients is also in accordance with the previous study conducted by Zhao and associates^17^.

Sjögren’s syndrome has been shown to negatively affect the periodontal condition^41^. Here, IP-10 was involved in the accumulation of T cell infiltration in the SS salivary gland^42^, whereas other studies have reported higher salivary concentration of MIP-1a in people with periodontal disease compared to healthy individuals^43,44^. In our study, we observed elevated levels of salivary IP 10 and MIP-1a in pSS patients, which correlated with certain dry mouth oral manifestations. In accordance, previous publications have also identified expression of IP-10 and MIPs in the salivary glands of pSS patients^42,45^.

A similar pattern of increase was observed for the anti-inflammatory cytokines IL-17 and IL-1ra in tear fluid of SS patients when compared to both healthy controls and non-SS sicca subjects. IL-17 is produced predominantly by Th17 cells, which have previously been detected in salivary glands and serum of SS patients, and is further associated with the pathogenesis of SS^46,47^. In addition, IL-17 may also play a role in corneal epithelial barrier disruption in dry eye disease^48^. Previous studies also showed a similar increase in IL-17 levels in tear fluid of SS patients^15,18^. Correspondingly, IL-1ra is a naturally occurring anti-inflammatory inhibitor of IL-1^49^, secreted mainly by monocytes and macrophages, as well as epithelial cells, in order to induce an endogenous regulatory mechanism against IL-1 mediated inflammation and tissue damage^50^. In ocular tissues, IL-1ra is also expressed abundantly in corneal and conjunctival epithelium^51–53^, where these elevated IL-1ra levels have been associated with reduced tear production measured with Schirmer’s test and increased conjunctival staining^54^. This may in turn be derived from inflammatory cells infiltrating the conjunctival epithelium^51^. Interestingly, our results also demonstrated increased levels of IL-1ra in the tear fluid of pSS patients compared to non-SS and healthy subjects, which were further associated with an increase of ocular surface staining. Taken together, our results indicate an increase of anti-inflammatory responses in the pSS patient group, as well as pro-inflammatory cytokine reactions. This further suggests interplay between pro- and anti-inflammatory reactions, in an attempt to establish a state of homeostasis in the pSS patient group.

Our present study also demonstrated that the severity of ocular surface damage, i.e. ocular surface and corneal staining in pSS, correlated with upregulated cytokines in tear fluid. This may represent a primary inflammatory effect of these cytokines on the surface epithelium. However, it may also be a secondary effect as a result of their ability to amplify inflammation by recruiting bone marrow derived cells to the ocular surface, which may consequently lead to the local production of cytokines at the site of inflammation. Interestingly, these elevated pro-inflammatory cytokines were only associated with reduced tear production, but not with reduced meibomian gland functionality and destructive morphological changes in pSS. Although meibomian gland dysfunction also contribute to dry eye disease in pSS, our results may imply that the reduced tear production is the main factor causing ocular surface inflammation.

In conclusion, we have demonstrated that upregulated cytokines identified in tear fluid and saliva of pSS patients show a clear interplay between innate and adaptive immune responses that may together further contribute to disease pathogenesis. Moreover, out of the 27 cytokines examined, the significant increase of IP-10 and MIP was found in both tears and saliva of pSS patients, further reflecting the essential role of macrophages and innate immunity in pSS. Whether these upregulated cytokines are a consequence of disease pathogenesis or a part of the repair process remains to be explored in follow-up studies.

## Supplementary information

Figure S1 and S2

## References

[1] Vitali C (2002). Classification criteria for Sjogren’s syndrome: a revised version of the European criteria proposed by the American-European Consensus Group. Annals of the rheumatic diseases.

[2] Fox PC (2007). Autoimmune diseases and Sjogren’s syndrome: an autoimmune exocrinopathy. Annals of the New York Academy of Sciences.

[3] Fox RI (2005). Sjogren’s syndrome. Lancet.

[4] Milin M (2016). Sicca symptoms are associated with similar fatigue, anxiety, depression, and quality-of-life impairments in patients with and without primary Sjogren’s syndrome. Joint, bone, spine: revue du rhumatisme.

[5] McMillan AS (2004). Impact of Sjogren’s syndrome on oral health-related quality of life in southern Chinese. Journal of oral rehabilitation.

[6] Hagan S, Tomlinson A (2013). Tear fluid biomarker profiling: a review of multiplex bead analysis. The ocular surface.

[7] Aota K (2018). Inverse correlation between the number of CXCR3(+) macrophages and the severity of inflammatory lesions in Sjogren’s syndrome salivary glands: A pilot study. Journal of oral pathology & medicine: official publication of the International Association of Oral Pathologists and the American Academy of Oral Pathology.

[8] Hillen MR, Ververs FA, Kruize AA, Van Roon JA (2014). Dendritic cells, T-cells and epithelial cells: a crucial interplay in immunopathology of primary Sjogren’s syndrome. Expert review of clinical immunology.

[9] Fox RI, Kang HI (1992). Pathogenesis of Sjogren’s syndrome. Rheumatic diseases clinics of North America.

[10] Aqrawi LA, Brokstad KA, Jakobsen K, Jonsson R, Skarstein K (2012). Low number of memory B cells in the salivary glands of patients with primary Sjogren’s syndrome. Autoimmunity.

[11] Chen W, Cao H, Lin J, Olsen N, Zheng SG (2015). Biomarkers for Primary Sjogren’s Syndrome. Genomics, proteomics & bioinformatics.

[12] Aqrawi LA (2017). Identification of potential saliva and tear biomarkers in primary Sjogren’s syndrome, utilising the extraction of extracellular vesicles and proteomics analysis. Arthritis Res Ther.

[13] Lee YJ (2010). Salivary chemokine levels in patients with primary Sjogren’s syndrome. Rheumatology.

[14] Kang EH, Lee YJ, Hyon JY, Yun PY, Song YW (2011). Salivary cytokine profiles in primary Sjogren’s syndrome differ from those in non-Sjogren sicca in terms of TNF-alpha levels and Th-1/Th-2 ratios. Clinical and experimental rheumatology.

[15] Lee SY (2013). Analysis of tear cytokines and clinical correlations in Sjogren syndrome dry eye patients and non-Sjogren syndrome dry eye patients. American journal of ophthalmology.

[16] Lopez-Miguel A (2016). Clinical and Molecular Inflammatory Response in Sjogren Syndrome-Associated Dry Eye Patients Under Desiccating Stress. American journal of ophthalmology.

[17] Zhao H, Li Q, Ye M, Yu J (2018). Tear Luminex Analysis in Dry Eye Patients. Medical science monitor: international medical journal of experimental and clinical research.

[18] Liu R (2017). Analysis of Th17-associated cytokines and clinical correlations in patients with dry eye disease. PloS one.

[19] Tashbayev B (2017). Interdisciplinary, Comprehensive Oral and Ocular Evaluation of Patients with Primary Sjogren’s Syndrome. Scientific reports.

[20] Chen X (2017). Meibomian gland features in a Norwegian cohort of patients with primary Sjogren’s syndrome. PloS one.

[21] Bron AJ, Evans VE, Smith JA (2003). Grading of corneal and conjunctival staining in the context of other dry eye tests. Cornea.

[22] The Definition and Classification of Dry Eye Disease: Report of the Definition and Classification Subcommittee of the International Dry Eye Workshop (2007). *The Ocular Surface* **5**, 75–92, 10.1016/S1542-0124(12)70081-2 (2007). 10.1016/s1542-0124(12)70081-2 17508116

[23] Thomson WM (2011). Shortening the xerostomia inventory. Oral surgery, oral medicine, oral pathology, oral radiology, and endodontics.

[24] Osailan SM, Pramanik R, Shirlaw P, Proctor GB, Challacombe SJ (2012). Clinical assessment of oral dryness: development of a scoring system related to salivary flow and mucosal wetness. Oral surgery, oral medicine, oral pathology and oral radiology.

[25] Landsend ECS (2018). The Level of Inflammatory Tear Cytokines is Elevated in Congenital Aniridia and Associated with Meibomian Gland Dysfunction. Investigative ophthalmology & visual science.

[26] Craig JP (2017). TFOS DEWS II Definition and Classification Report. The ocular surface.

[27] Stern ME (2002). Conjunctival T-cell subpopulations in Sjogren’s and non-Sjogren’s patients with dry eye. Investigative ophthalmology & visual science.

[28] Jones DT, Monroy D, Ji Z, Atherton SS, Pflugfelder SC (1994). Sjogren’s syndrome: cytokine and Epstein-Barr viral gene expression within the conjunctival epithelium. Investigative ophthalmology & visual science.

[29] Szodoray P (2008). Immunological alterations in newly diagnosed primary Sjogren’s syndrome characterized by skewed peripheral T-cell subsets and inflammatory cytokines. Scandinavian journal of rheumatology.

[30] Brookes SM, Price EJ, Venables PJ, Maini RN (1995). Interferon-gamma and epithelial cell activation in Sjogren’s syndrome. British journal of rheumatology.

[31] Pflugfelder SC (2015). Aqueous Tear Deficiency Increases Conjunctival Interferon-gamma (IFN-gamma) Expression and Goblet Cell Loss. Investigative ophthalmology & visual science.

[32] Zhang X (2011). Desiccating stress induces CD4+ T-cell-mediated Sjogren’s syndrome-like corneal epithelial apoptosis via activation of the extrinsic apoptotic pathway by interferon-gamma. The American journal of pathology.

[33] Trinchieri G (2003). Interleukin-12 and the regulation of innate resistance and adaptive immunity. Nature reviews. Immunology.

[34] Kohriyama K, Katayama Y (2000). Disproportion of helper T cell subsets in peripheral blood of patients with primary Sjogren’s syndrome. Autoimmunity.

[35] Koarada S (2006). *Ex vivo* CD(+) T-cell cytokine expression from patients with Sjogren’s syndrome following *in vitro* stimulation to induce proliferation. Rheumatology.

[36] Choi W (2012). Expression of CCR5 and its ligands CCL3, -4, and -5 in the tear film and ocular surface of patients with dry eye disease. Current eye research.

[37] Gulati A, Sacchetti M, Bonini S, Dana R (2006). Chemokine receptor CCR5 expression in conjunctival epithelium of patients with dry eye syndrome. Archives of ophthalmology.

[38] Yoon KC (2010). Expression of CXCL9, -10, -11, and CXCR3 in the tear film and ocular surface of patients with dry eye syndrome. Investigative ophthalmology & visual science.

[39] Larsen C (1990). Proinflammatory cytokines interleukin 1 and tumor necrosis factor induce cytokines that are chemotactic for neutrophils, T cells and monocytes. Progress in clinical and biological research.

[40] Erger RA, Casale TB (1995). Interleukin-8 is a potent mediator of eosinophil chemotaxis through endothelium and epithelium. The American journal of physiology.

[41] Antoniazzi RP (2009). Periodontal conditions of individuals with Sjogren’s syndrome. Journal of periodontology.

[42] Ogawa N, Ping L, Zhenjun L, Takada Y, Sugai S (2002). Involvement of the interferon-gamma-induced T cell-attracting chemokines, interferon-gamma-inducible 10-kd protein (CXCL10) and monokine induced by interferon-gamma (CXCL9), in the salivary gland lesions of patients with Sjogren’s syndrome. Arthritis and rheumatism.

[43] Al-Sabbagh M (2012). Bone remodeling-associated salivary biomarker MIP-1alpha distinguishes periodontal disease from health. Journal of periodontal research.

[44] Syndergaard B (2014). Salivary biomarkers associated with gingivitis and response to therapy. Journal of periodontology.

[45] Cuello C (1998). Chemokine expression and leucocyte infiltration in Sjogren’s syndrome. British journal of rheumatology.

[46] Sakai A, Sugawara Y, Kuroishi T, Sasano T, Sugawara S (2008). Identification of IL-18 and Th17 cells in salivary glands of patients with Sjogren’s syndrome, and amplification of IL-17-mediated secretion of inflammatory cytokines from salivary gland cells by IL-18. Journal of immunology.

[47] Oh JY (2011). Investigating the relationship between serum interleukin-17 levels and systemic immune-mediated disease in patients with dry eye syndrome. Korean journal of ophthalmology: KJO.

[48] De Paiva CS (2009). IL-17 disrupts corneal barrier following desiccating stress. Mucosal immunology.

[49] Dinarello CA (2009). Interleukin-1beta and the autoinflammatory diseases. The New England journal of medicine.

[50] Arend WP (1993). Interleukin-1 receptor antagonist. Advances in immunology.

[51] Solomon A (2001). Pro- and anti-inflammatory forms of interleukin-1 in the tear fluid and conjunctiva of patients with dry-eye disease. Investigative ophthalmology & visual science.

[52] Gamache DA (1997). Secretion of proinflammatory cytokines by human conjunctival epithelial cells. Ocular immunology and inflammation.

[53] Kennedy MC (1995). Novel production of interleukin-1 receptor antagonist peptides in normal human cornea. The Journal of clinical investigation.

[54] Huang JF, Zhang Y, Rittenhouse KD, Pickering EH, McDowell MT (2012). Evaluations of tear protein markers in dry eye disease: repeatability of measurement and correlation with disease. Investigative ophthalmology & visual science.

