# Supplementary material for: Elevated cytokine levels in tears and saliva of patients with primary Sjögren’s syndrome correlate with clinical ocular and oral manifestations
Source: Sci Rep. 2019 May 13;9:7319. doi: 10.1038/s41598-019-43714-5 (PMC6513950; doi:10.1038/s41598-019-43714-5)
Supplement: Supplementary file 1 — Figure S1 and S2 [file 41598_2019_43714_MOESM1_ESM.pdf]

Supplementary Figures S1 and S2

Manuscript title: **Elevated cytokine levels in tears and saliva of patients with primary Sjögren's syndrome correlate with clinical ocular and oral manifestations**

Xiangjun Chen[1,2*], Lara A. Aqrawi[1], Tor Paaske Utheim[2,3,4], Behzod Tashbayev[1,2], Øygunn Aass Utheim[2], Sjur Reppe[4], Lene Hystad Hove[5], Bente Brokstad Herlofson[1], Preet Bano Singh[1], Øyvind Palm[6], Hilde Kanli Galtung[3], Janicke Cecilie Liaaen Jensen[1]

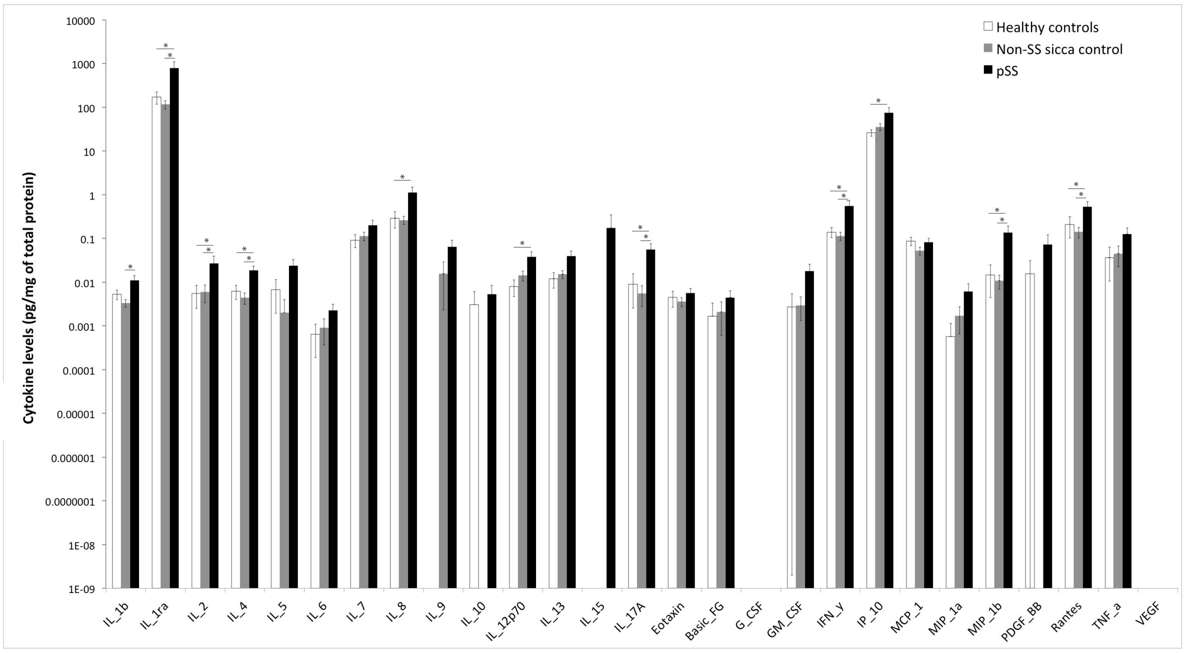

Figure S1. **Comparison of tear cytokine levels adjusted by total protein.** Intergroup difference is considered significant at *$p < 0.05$.

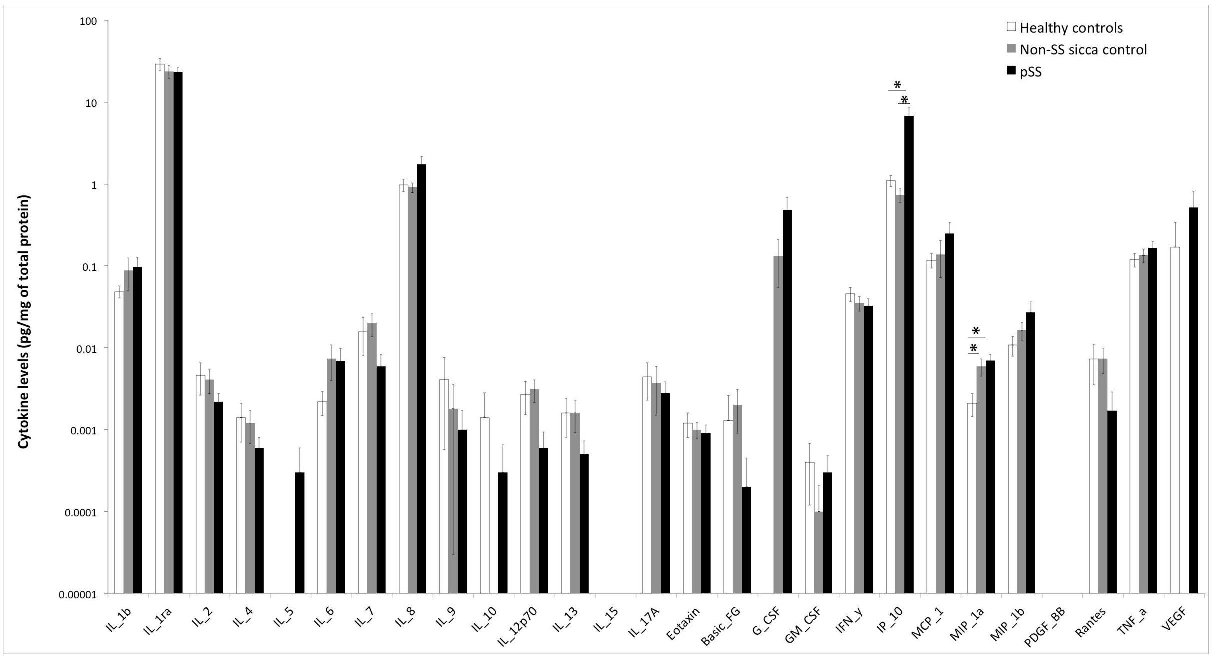

Figure S2. **Comparison of salivary cytokine levels adjusted by total protein.** Intergroup difference is considered significant at *$p < 0.05$.
